# Advancing Alzheimer's research: Radiomics visualization of the default mode network in cerebral perfusion imaging

**DOI:** 10.1002/acm2.14368

**Published:** 2024-04-24

**Authors:** Danzhou Fang, Zhiming Zhou, Yalan Xiong, Yongzeng Fan, Yixuan Li, Huayi Zhao, Jiahui Huang, Gengbiao Yuan, Maohua Rao

**Affiliations:** ^1^ Department of Nuclear Medicine Second Affiliated Hospital of Chongqing Medical University Chongqing China; ^2^ Department of Radiology Second Affiliated Hospital of Chongqing Medical University Chongqing China

**Keywords:** Alzheimer's disease, Cerebral blood flow perfusion imaging, Default mode network (DMN), Radiomics

## Abstract

**Objective:**

Alzheimer's disease, an irreversible neurological condition, demands timely diagnosis for effective clinical intervention. This study employs radiomics analysis to assess image features in default mode network cerebral perfusion imaging among individuals with cognitive impairment.

**Methods:**

A radiomics analysis of cerebral perfusion imaging was conducted on 117 patients with cognitive impairment. They were divided into training and validation sets in a 7:3 ratio. Least Absolute Shrinkage and Selection Operator (LASSO) and Random Forest were employed to select and model image features, followed by logistic regression analysis of LASSO and Random Forest results. Diagnostic performance was assessed by calculating the area under the curve (AUC).

**Results:**

In the training set, LASSO achieved AUC of 0.978, Random Forest had an AUC of 0.933. In the validation set, LASSO had AUC of 0.859, Random Forest had AUC of 0.986. By conducting Logistic Regression analysis in combination with LASSO and Random Forest, we identified a total of five radiomics features, with four related to morphology and one to textural features, originating from the medial prefrontal cortex and middle temporal gyrus. In the training set, Logistic Regression achieved AUC of 0.911, while in the validation set, it attained AUC of 0.925.

**Conclusion:**

The medial prefrontal cortex and middle temporal gyrus are the two brain regions within the default mode network that hold the highest significance for Alzheimer's disease diagnosis. Radiomics analysis contributes to the clinical assessment of Alzheimer's disease by delving into image data to extract deeper layers of information.

## 1 INTRODUCTION

Alzheimer's disease (AD) is an irreversible condition characterized by memory loss and cognitive dysfunction, representing the most common cause of dementia.^1^ By 2050, an estimated 44 million people worldwide are projected to experience AD.^2^ Despite significant progress in understanding AD's pathogenesis and conceptualization, effective treatments for AD patients have remained elusive.^3^ Determining with precision whether patients with declining cognitive function have Alzheimer's Disease (AD) is of paramount importance. Single‐photon emission computed tomography (SPECT) is a reliable method for diagnosing AD, commonly used to assess regional cerebral blood flow.^4^ Brain SPECT is a non‐invasive examination method has been demonstrated to be effective in identifying AD and non‐AD patients.^5^ Previous studies have shown that a decrease in blood perfusion in the parietal temporal lobe and posterior cingulate gyrus on SPECT can be an early detection sign of AD.^6^ In AD patients, SPECT imaging can detect a reduction in blood flow signals in certain brain regions associated with the Default Mode Network (DMN) mechanism. DMN is a specific neural network in the brain that is associated with cognitive processes related to self‐reflection, introspection, memory recall, and inner thoughts and emotions. The activity of this network is typically more active during a person's resting state and less active when engaged in cognitive tasks. The DMN comprises several key regions, including the medial prefrontal cortex, posterior cingulate cortex, precuneus, middle temporal gyrus, and bilateral angular gyrus.^7^ However, interpreting SPECT images for AD diagnosis can be subjective and may vary among nuclear medicine physicians, making it challenging to accurately identify subtle changes in brain perfusion among individuals with cognitive impairment.

To address this issue, radiomics analysis of cerebral perfusion imaging offers a promising avenue.^8^ Radiomics allows for the extraction of image features and subsequent data mining in an automated, objective manner. By analyzing these image features in depth, radiomics can aid in the precise and early identification of AD, focusing on local texture and global measurements.^9^, ^10^ Currently, research primarily focuses on radiomics analysis of MRI images in AD patients, and there is relatively limited research on SPECT cerebral perfusion imaging. This study aims to use radiomics analysis to evaluate the image features of DMN cerebral perfusion imaging in patients with cognitive impairment, seeking to identify differences in radiomics features between AD and non‐AD patients and provide further evidence for the diagnosis of AD.

## 2 MATERIALS AND METHODS

### 2.1 Patients

This study involved 117 patients with cognitive impairment who were admitted to our hospital between September 2017 and September 2022. This retrospective study, approved by ethics committee of our hospital (No. 2023.72), was conducted with no need for written informed consent. Admitted patients met the following inclusion criteria: 1) memory loss; 2) cognitive dysfunction; 3) cognitive impairment in one or more cognitive domains including attention, language, visual space, and executive ability. Exclusion criteria included: 1) cognitive impairment caused by head trauma or craniocerebral surgery; 2) current or lifelong history of neurological or mental illness that may lead to cognitive impairment, such as stroke, depression, or epilepsy; 3) nervous system defects, such as vision or hearing loss; and 4) a history of serious heart, lung, liver, or kidney disease. The diagnosis of AD was based on the criteria established by the National Institute of Neurological and Communication Disorders and Stroke and the Alzheimer's Disease and Related Disorders Association.^11^

Each patient underwent the same standardized procedure. After the intravenous injection of 20mCi ^99 m^Tc‐ECD in a dark and quiet room without ear plugging for 30 min, each patient underwent scanning using the identical machine (GE Millenium VG). Each patient's head was secured to a special head bracket and secured with a strap, allowing the camera detector to rotate close to their head. The image was recorded on a 128 × 128 matrix in a step and shoot mode (128 projections, 30 s per projection). Reconstruction was done through the use of a filtered back projection technique, and a universal Wiener filter was used for smoothing. Before extracting features, the SPECT images were normalized by centering around the mean of the standard deviation to mitigate the adverse effects of abnormal gray values.

### 2.2 Classification model

#### 2.2.1 Feature extraction

Two nuclear medicine physicians with over 5 years of experience manually delineated the Regions of Interest (ROIs) in the DMN on axial brain sections using ITK‐SNAP software (version 3.8.0).ROIs are manually segmented according to the DMN partition.^7^ They worked separately, without interference or communication, and maintained blindness to patient information during the delineation process. Subsequently, a senior nuclear medicine physician with more than 15 years of clinical experience reviewed the results. Pyradiomics software (version 3.0.1) was employed to automatically extract 107 radiomics features from each ROI, categorizing these features into seven distinct classes.

#### 2.2.2 Feature selection and classification models

**FIGURE 1:**
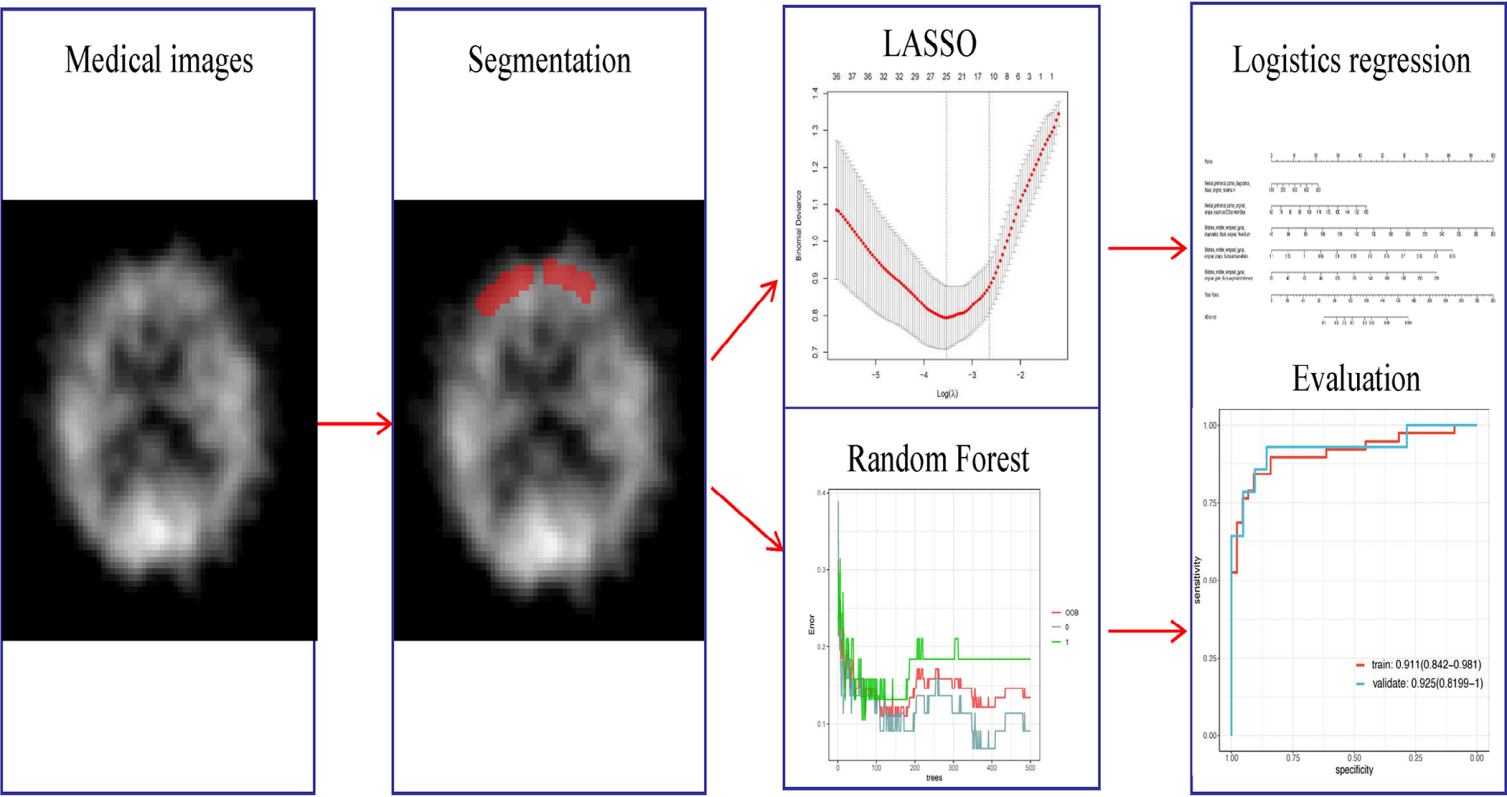
Process of constructing LASSO and Random Forest models. The flowchart effectively illustrates the radiomics workflow of cerebral blood flow perfusion imaging.

To screen out the key features, LASSO Glmnet' (Glmnet 4.1‐6) and Random Forest (random forest 4.7‐1.1) were employed to analyze selected candidate image features and calculate the importance of key features in the dataset. Patients were randomly divided into a training set (*n* = 82) and a validation set (*n* = 35) in a 7:3 ratio. *Z*‐score normalization was employed to mitigate the dimensional effects of different features. LASSO with 5‐fold cross‐validation was used to extract the most relevant features for AD diagnosis from the 107 available features in each ROI.^12^ In this study, we provide an illustration of the construction process for LASSO and Random Forest models, as depicted in Figure 1. Two models were further verified using the receiver operating characteristic curve by employing the “ROCR” package (version 1.18.0). Using the LASSO and Random Forest models, a logistic regression analysis was employed to identify the more valuable imaging features for AD diagnosis. The logistic regression analysis model is visualized using a nomogram, and after ROC analysis, the AUC value for diagnosing AD is calculated. The calibration curve was then used to evaluate the goodness of fit of the logistic regression in distinguishing AD.

### 2.3 Statistical analysis

In statistical tests of demographical and clinical characteristics, the Chi‐square test and rank sum test (SPSS 26.0) were employed to analyze numerical variables. *p‐*value less than 0.05 was deemed statistically significant. To evaluate the performance of the model, the AUC was assessed.^13^ The Hosmer‐Lemeshow test (ResourceSelection 0.3‐5) was used to evaluate the fitting degree of the Logistic Regression, while the root mean square (RMS 6.5‐0) was used to generate the calibration curve.

## 3 RESULTS

### 3.1 Basic patient characteristics

Table 1 shows that there is no significant difference in age, gender, or years of cognitive decline between the AD and no‐AD groups. However, there is a statistically significant difference in Mini‐Mental State Examination scores between the AD and no‐AD groups (*p* = 0.008).

**TABLE 1:** Baseline characteristics of training cohort and validation cohort.

Name	Levels	non‐AD (*N* = 65)	AD (*N* = 52)	Total (*N* = 117)	*p*
Gender	Male	22 (33.8%)	20 (38.5%)	42 (35.9%)	0.747
	Female	43 (66.2%)	32 (61.5%)	75 (64.1%)	
Age	Median (IQR)	70.0 (64.0–78.0)	69.0 (63.0–79.0)	70.0 (64.0–78.0)	0.858
Cognitive disorder	Median (IQR)	2.0 (0.5–3.0)	2.0 (1.0–3.0)	2.0 (1.0–3.0)	0.24

### 3.2 LASSO model

**FIGURE 2:**
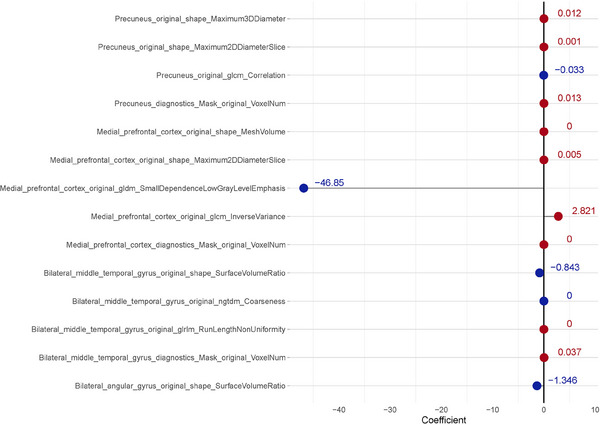
LASSO was used for feature selection. Using 5‐fold cross validation,14 nonzero coefficients were selected. The larger the coefficient, the more relevant the AD diagnosis was.

Figure S1 shows the LASSO filter process diagram. As shown in Figure 2, LASSO selected the most important 14 features from a total of 62595 features. The larger the absolute value, the more valuable the feature is for AD diagnosis. A total of 14 radiomics features were ultimately selected (as shown in Figure 2). Among these features, nine belong to morphology, two are related to image intensity, and three pertain to textural features. These 14 features were extracted from the medial prefrontal cortex, precuneus, middle temporal gyrus, and bilateral angular gyrus.

### 3.3 Random forest model

**FIGURE 3:**
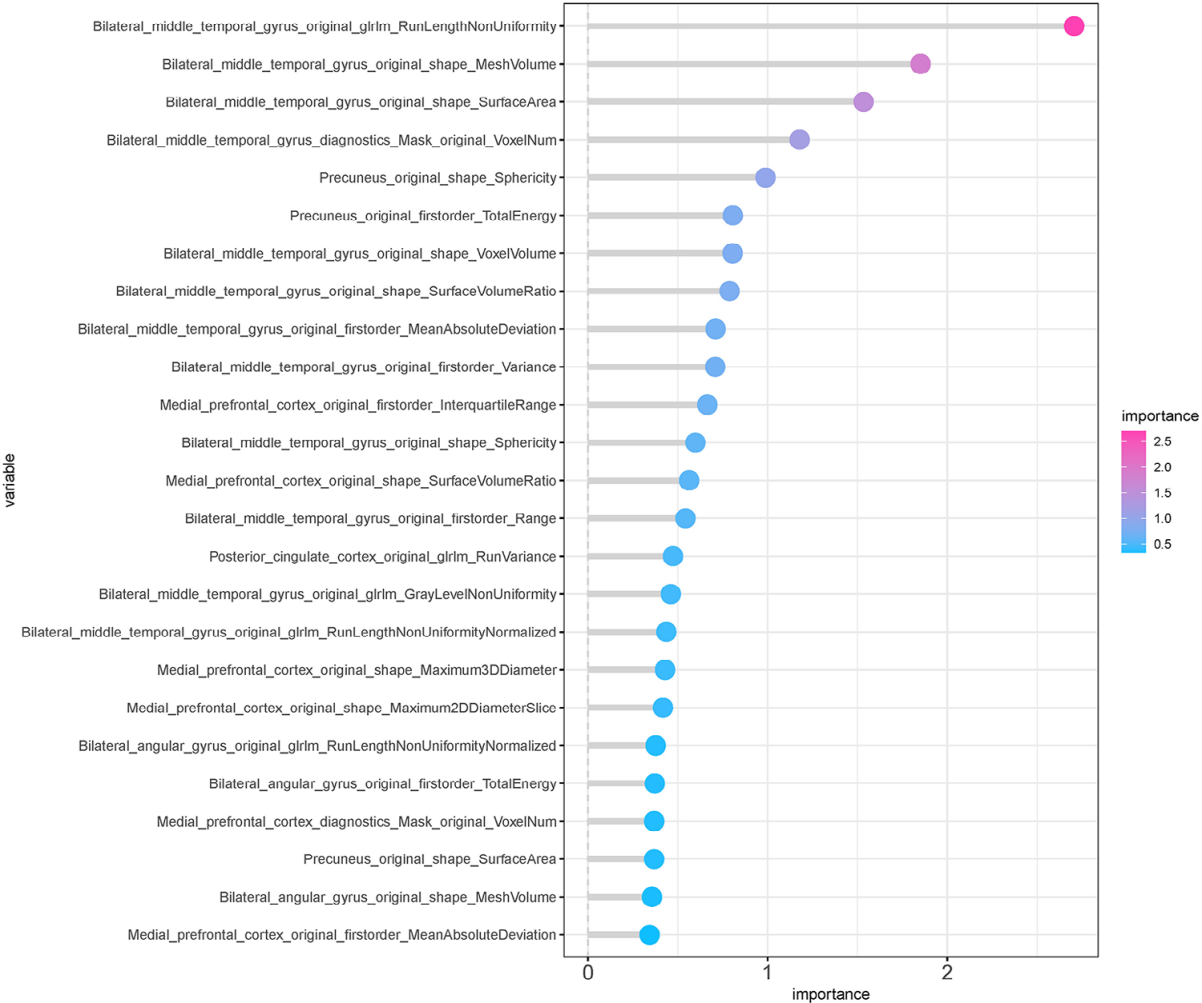
The importance of image features extracted from random forest. In the random forest model, the greater the value, the greater the effect on the diagnosis of AD.

Figure S2 shows the Random Forest filter process diagram. As shown in Figure 3, the significance of image features derived from the Random Forest model. A higher value within the Random Forest model indicates a stronger influence on AD diagnosis. A total of 25 radiomics features were eventually chosen through the utilization of Random Forest, as illustrated in Figure 3. Within this selection, 12 are associated with morphology, 8 are connected to image intensity, and 5 are related to textural features. These 25 features were derived from the medial prefrontal cortex, precuneus, middle temporal gyrus, and bilateral angular gyrus.

### 3.4 Evaluation of LASSO and random forest model

**FIGURE 4:**
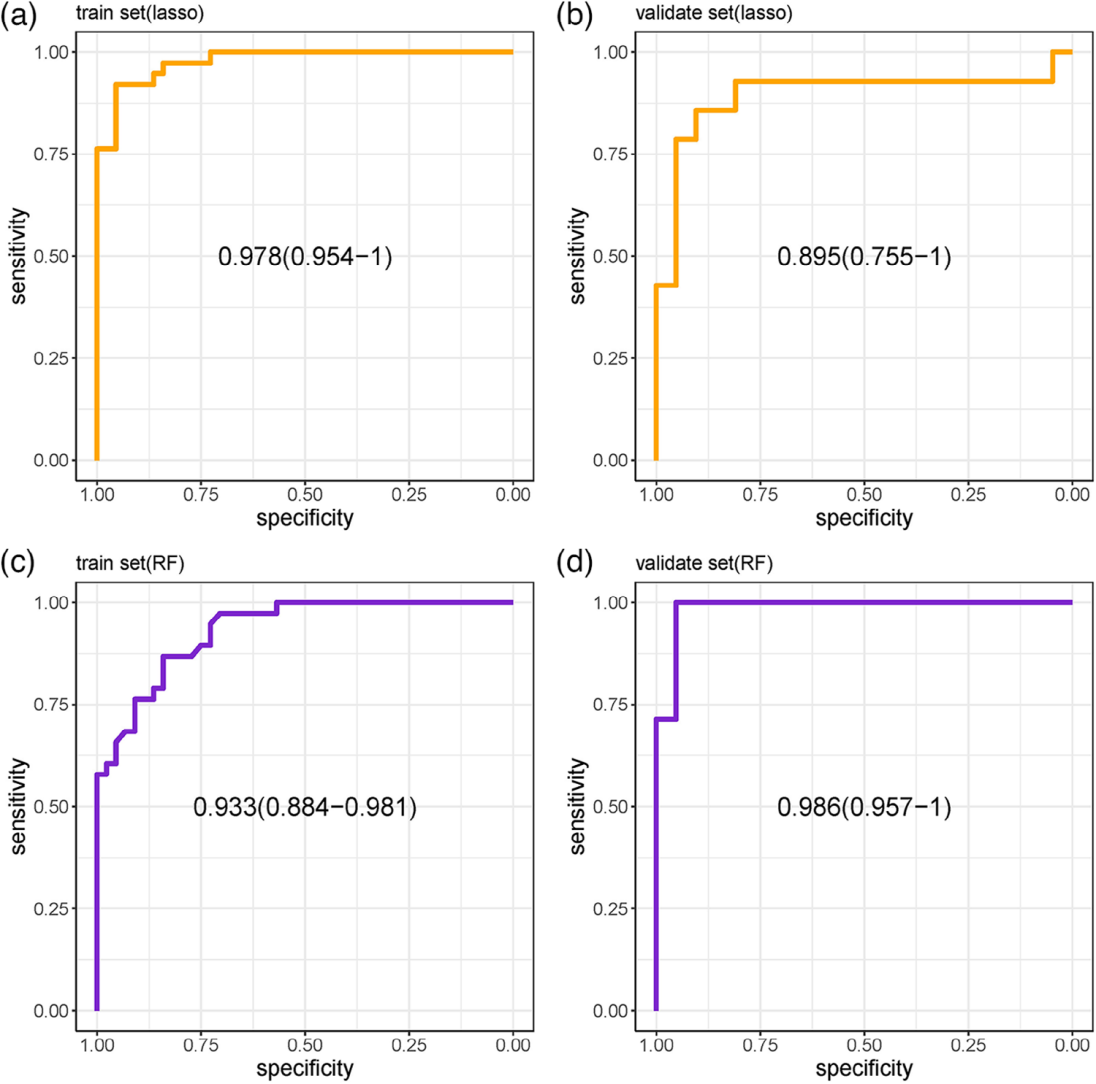
AUC of LASSO (a and b) and random forest (c and d). In the LASSO model's training cohort, the AUC was 0.978 ([CI] 0.954–1). In the validation cohort of the LASSO model, the AUC was 0.859 ([CI] 0.755–1). In the training cohort, the RF model demonstrated an AUC of 0.933 ([CI] 0.884–0.981) for diagnosing AD, while in the validation cohort, the RF model had an AUC of 0.986 ([CI] 0.957–1) for AD diagnosis.

From Figure 4a,b it can be observed that in the LASSO training cohort, the AUC was 0.978 (95% CI: 0.954–1.000), with a specificity of 0.955, sensitivity of 0.921, and accuracy of 0.939. In the validation cohort, the AUC was 0.859 (95% CI: 0.755−1.000), with a specificity of 0.905, sensitivity of 0.857, and accuracy of 0.886. Figure 4c,d reveals that within the Random Forest training cohort, the AUC measured 0.933 (95% CI: 0.884−0.981) with a specificity of 0.841, a sensitivity of 0.868, and an accuracy of 0.854. Meanwhile, the validation cohort showed an AUC of 0.986 (95% CI: 0.957−1.000), complemented by a specificity of 0.886, a sensitivity of 1, and an accuracy of 0.971. Both the LASSO and Random Forest models demonstrated good diagnostic performance.

### 3.5 Logistic regression

**FIGURE 5:**
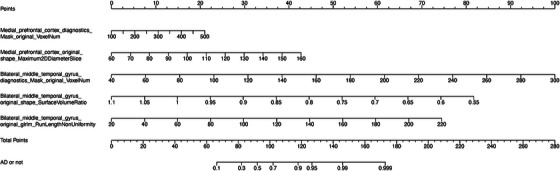
Logistics regression of the identifying AD. This nomogram illustrates the five most significant features derived from radiomics. Each feature is assigned a corresponding score, contributing to the overall predictive model used to differentiate AD and non‐AD patients. The scores associated with each feature represent their respective impact on the predictive outcome.

**FIGURE 6:**
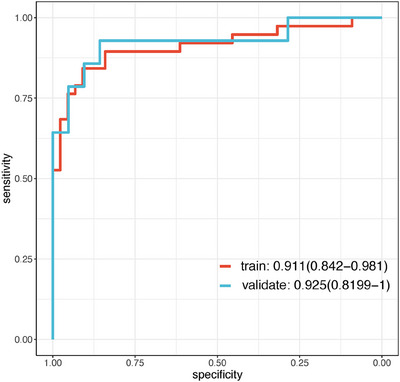
AUC OF logistics regression. The AUC of the logistics regression was 0.911 (95% CI:0.842–0.981) in the training set and 0.925 (95% CI:0.819–1) in the validation set.

**FIGURE 7:**
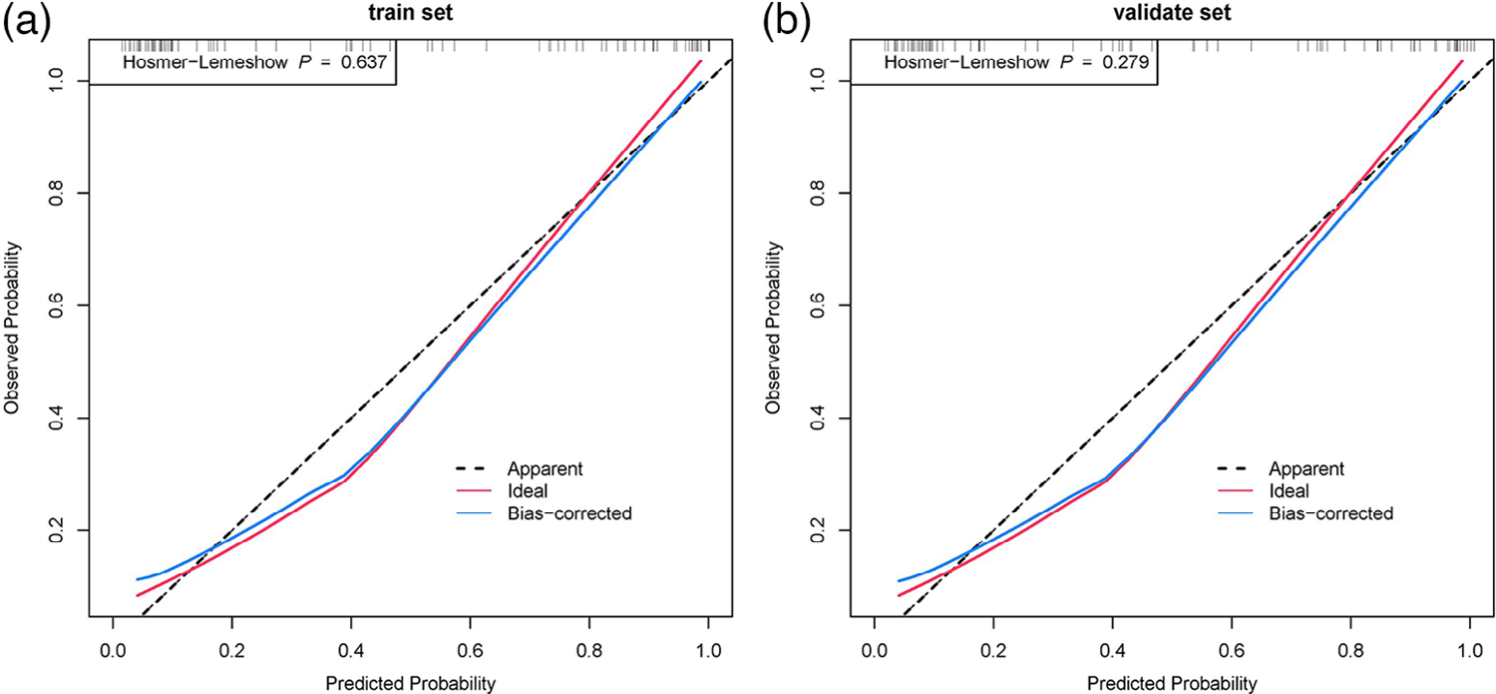
The calibration curve of logistics regression. In the calibration curve of logistics regression, the *p*‐value of the training set is 0.637, and the *p*‐value of the validation set is 0.279.

Logistic regression analysis was performed on the screening results of LASSO and random forest to further screen imaging features. Finally, five radiomics features were screened out, as shown in Figure 5. In this set, four features are linked to morphology, while one pertains to textural features. These five features were extracted from the medial prefrontal cortex and bilateral middle temporal gyrus. In the training cohort, the Logistic Regression model demonstrated an AUC of 0.911 (95% CI, 0.842–0.981), with a specificity of 0.909, sensitivity of 0.842, and an accuracy of 0.878. For the validation cohort, the model achieved an AUC of 0.925 (95% CI, 0.8199–1), with a specificity of 0.857, sensitivity of 0.929, and an accuracy of 0.886, as depicted in Figure 6. The training set and validation set of the logistic regression analysis both passed the Hosmer‐Lemeshow test, as indicated by *p*‐values of 0.637 for the training set and 0.279 for the validation set, as observed from Figure 7. Overall, the results of this study indicate that the medial prefrontal cortex and middle temporal gyrus are the most influential factors in the DMN network structure for AD diagnosis.

## 4 DISCUSSION

Radiomics proves to be a valuable tool in disease management, offering a multi‐faceted analysis of medical images.^14^ In our study, we conducted a comprehensive analysis of various microstructural features within the DMN ROIs, covering aspects such as morphology, signal intensity, and textural features.^7^ Due to the substantial overlap of the DMN with AD pathological regions, the DMN has been a focal point in AD biomarker research.^15^ When the brain is at rest, specific active regions form the DMN.^16^ The DMN is associated with various cognitive processes, including consciousness, episodic memory, environmental recognition, and emotion.^17^ AD patients exhibit significant alterations in the DMN, along with the salience network.^18^ Common clinical symptoms of AD, such as reduced cognitive function and episodic memory, are often linked to DMN dysfunction.^19^, ^20^ The DMN comprises several key regions, including the medial prefrontal cortex, posterior cingulate cortex, precuneus, middle temporal gyrus, and bilateral angular gyrus.^7^ Medial temporal lobe atrophy is considered one of the signs of AD,^21^ and the medial prefrontal cortex has been found to play a role in self‐reference thinking including introspection, self‐evaluation, social cognition, and processing of self and other related information.^22^ The posterior cingulate cortex is involved in a multitude of cognitive processes, such as memory retrieval, navigation, self‐processing, attention regulation, and alertness.^23^ The middle temporal gyrus region of the brain is involved in many cognitive functions, such as language processing, auditory perception, semantic memory, social cognition,^24^ constructing coherent scenarios, and forming events,^25^ and mental models.^26^ In AD patients, senile plaques and neurofibrillary tangles can be observed in the middle temporal gyrus of the DMN system.^27^ The function of the bilateral angular gyrus may be related to compensating for deficits in memory coding.^28^

Cerebral blood flow, closely linked to brain function and activity, refers to the supply of blood to the brain through the cerebral vascular system. The brain requires an adequate supply of blood and oxygen to maintain normal functionality. When different regions of the brain become active, blood flow increases correspondingly to meet the demands of these regions. Cerebral blood flow can be monitored through cerebral blood flow perfusion imaging to study the activity and changes in blood flow within specific brain areas, helping to uncover the mechanisms behind brain function and diseases.^29^ SPECT studies have shown a reduction in regional cerebral blood flow in the frontal lobe, temporal lobe, and parietal lobe, with a milder reduction observed in the parietal and occipital lobes.^30^

In LASSO models, the importance of features is determined by their coefficients, where the magnitude of the coefficients reflects the degree of influence on the target variable. By adding an L1 penalty, LASSO promotes sparsity in the model, shrinking the coefficients of features with lesser impact on the target variable toward zero, thereby excluding them. In contrast, in random forest models, feature importance is determined by evaluating their contribution to reducing errors within the decision tree ensemble. Because random forests can consider the complex relationships between features more comprehensively, some features deemed unimportant and excluded in LASSO may still have significant impact on the model's accuracy in random forests, and vice versa. This implies that feature selection may differ between the two models, resulting in different features being retained or excluded. In the LASSO model, a total of 14 distinct features were considered, also encompassing morphological, image intensity, and textural characteristics. These features were derived from the same brain regions mentioned earlier. In the LASSO model, nine features were related to morphology, two to image intensity, and three to textural features. Within Random Forest model selection, a total of 25 features were considered, encompassing morphological, image intensity, and textural characteristics. These features were derived from various brain regions, including the medial prefrontal cortex, precuneus, middle temporal gyrus, and bilateral angular gyrus. Specifically, in the Random Forest model, 12 features were associated with morphology, 8 with image intensity, and 5 with textural features. Using LASSO or random forest models, we did not observe a decrease in regional cerebral blood flow in the Posterior Cingulate Cortex and the precuneus. These brain regions are situated between the parietal lobe and occipital lobe. Importantly, our study found no significant differences between the group of patients with AD and those without AD in these specific brain regions. There are some limitations in this study. First of all, the segmentation of ROI depends on nuclear medicine experts, which is easy to cause deviation. The most prominent reason why morphological (shape‐based) features are prominent in the list of important features is that the ROI is quite small and contains only a limited number of pixels. Secondly, the resolution of SPECT is not as good as PET. Third, the emergence of new imaging agents, such as ^18^F‐FDG, can provide glucose metabolism information for the diagnosis of AD. Finally, The study's sample size is relatively small, and it is sourced from the same center with the same scanning machine. Further investigation is required with a larger, multi‐center sample size to validate the findings. Our study found hypoperfusion in the middle temporal gyrus of AD patients, which is consistent with previous reports indicating decreased hypoperfusion of the middle temporal gyrus can be used as an auxiliary diagnosis of AD.^31^ Through logistic regression, five radiomics features were selected, with four related to morphology and one related to textural features. Even if the AUC value of Logistic Regression is not as high as that of Random Forest, it still holds significance. Logistic Regression is a simple and interpretable model suitable for modeling and predicting linear relationships. It has advantages in interpretability, clearly illustrating the impact of features on the target variable. Additionally, Logistic Regression models typically have lower computational costs and faster training speeds, making them suitable for handling large‐scale datasets. Each voxel contains an intensity value for each channel of the image. Shape‐based features such as 2D and 3D diameters, axes, and their ratios can be used to describe geometric properties of ROIs.^32^ GLRLM, a radiomics‐related feature related to texture analysis, can be used to reflect the roughness and complexity between different pixels in the middle temporal gyrus.^33^ Regional gray matter, which is linked to psychological ability and social network size,^34^ has also been correlated with the structure of the DMN network for AD diagnosis. In AD patients, significant differences in gray matter volume in the medial temporal region suggest that decreased perfusion may result from remote brain region remodeling or compensatory middle temporal gyrus activation.^35^ The metabolic hypothesis posits that continuous DMN activity may lead to a metabolic cascade contributing to AD pathology.^36^ Increased prefrontal recruitment compensates for reduced cognitive resources.^37^ Additionally, the medial frontal area plays a broader role in social cognition, empathy processes, and tasks related to detecting empathy and autobiographical memory, requiring self‐referential processing, but is also common in the task of detecting empathy.^38^, ^39^ The medial prefrontal cortex is crucial in extracting, organizing, and integrating autobiographical memories.^40^ Our study found that the medial prefrontal cortex and bilateral middle temporal regions exhibit the most significant differences between AD and non‐AD patients.

## 5 CONCLUSION

Through the utilization of LASSO, Random Forest, and selected features, our study ultimately identified the radiomics features of medial prefrontal cortex and middle temporal gyrus as the most influential factors in the DMN network structure for AD diagnosis. Delving deeper into information extraction, Radiomics analysis aids in providing more evidence for the diagnosis of the AD group. It involves the extraction and analysis of numerous quantitative features from medical images to gather information. Radiomics has the potential to be a vital tool in personalized medicine, offering additional insights for disease identification and patient management.

## AUTHOR CONTRIBUTIONS

Danzhou Fang wrote the manuscript. Zhiming Zhou, Yalan Xiong, and Yongzeng Fan provided technical assistance. Yixuan Li, Huayi Zhao, and Jiahui Huang collected data. Maohua Rao and Gengbiao Yuan reviewed the manuscript.

## CONFLICT OF INTEREST STATEMENT

The authors declare no conflicts of interest.

## Supporting information

Supporting Information

## Data Availability

Authors will share data upon request to the corresponding author.
