# Supplementary material for: Advancing Alzheimer's research: Radiomics visualization of the default mode network in cerebral perfusion imaging
Source: J Appl Clin Med Phys. 2024 Apr 24;25(5):e14368. doi: 10.1002/acm2.14368 (PMC11087173; doi:10.1002/acm2.14368)
Supplement: Supplementary file 1 — Supporting Information [file ACM2-25-e14368-s001.docx]

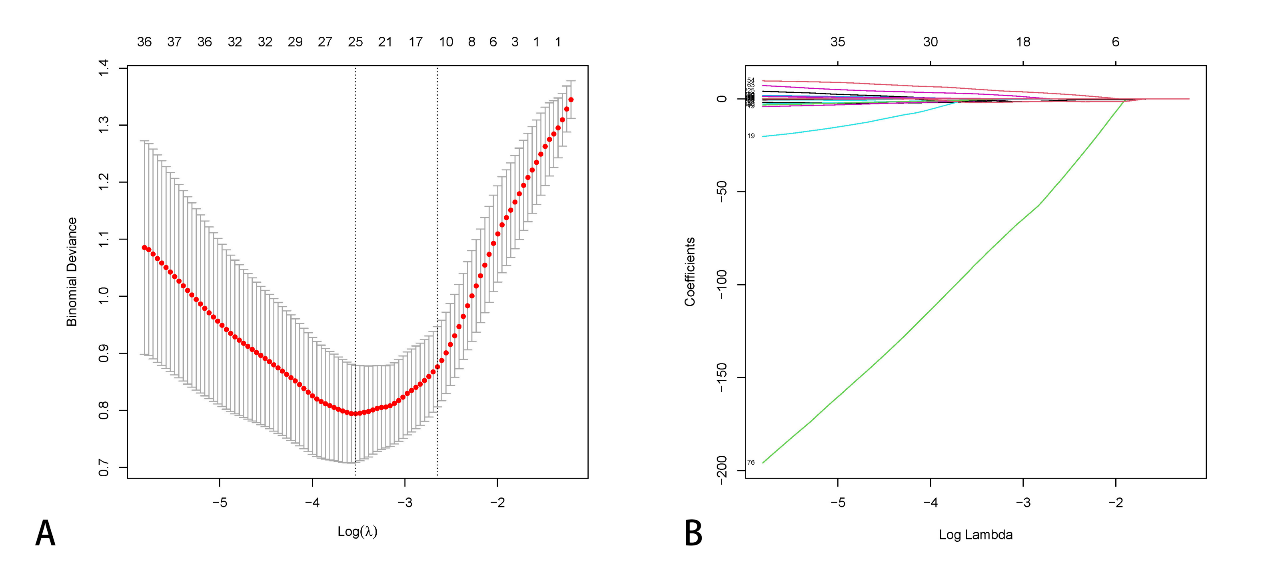
Supplementary Fig.1: LASSO is used for feature selection.

A. LASSO was used to select the radiomics features that could diagnose AD. Tuning parameter selection in LASSO. B. 14 coefficients were selected.


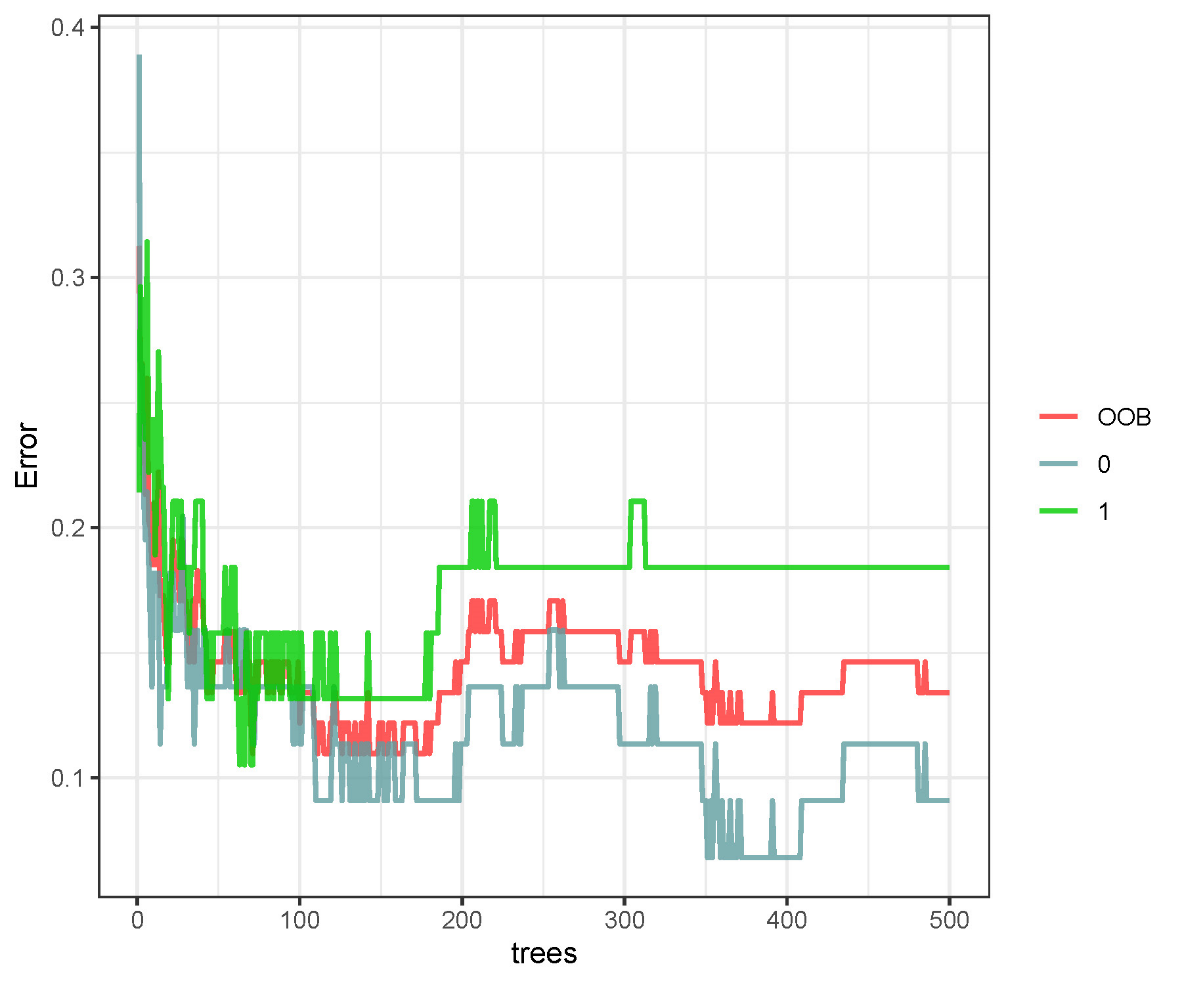


Supplementary Fig.2:Random forest for feature selection. Number of decision trees and the average out-of-error rate when establishing a random forest model.
